# CO_2_ Methanation: Solvent-Free Synthesis of Nickel-Containing Catalysts from Complexes with Ethylenediamine

**DOI:** 10.3390/ma16072616

**Published:** 2023-03-25

**Authors:** Olga V. Netskina, Kirill A. Dmitruk, Olga I. Mazina, Alexander A. Paletsky, Svetlana A. Mukha, Igor P. Prosvirin, Alena A. Pochtar, Olga A. Bulavchenko, Andrey G. Shmakov, Janna V. Veselovskaya, Oxana V. Komova

**Affiliations:** 1 Boreskov Institute of Catalysis SB RAS, Pr. Akademika Lavrentieva 5, 630090 Novosibirsk, Russia; 2 Department of Natural Sciences, Novosibirsk State University, 1 Pirogova Str., 630090 Novosibirsk, Russia; 3 Voevodsky Institute of Chemical Kinetics and Combustion SB RAS, 3 Institutskaya Str., 630090 Novosibirsk, Russia

**Keywords:** CO_2_ methanation, nickel, ethylenediamine, complex, solvent-free synthesis, solid-state combustion

## Abstract

CO_2_ methanation was studied in the presence of nickel catalysts obtained by the solid-state combustion method. Complexes with a varying number of ethylenediamine molecules in the coordination sphere of nickel were chosen as the precursors of the active component of the catalysts. Their synthesis was carried out without the use of solvents, which made it possible to avoid the stages of their separation from the solution and the utilization of waste liquids. The composition and structure of the synthesized complexes were confirmed by elemental analysis, IR spectroscopy, powder XRD and XPS methods. It was determined that their thermal decomposition in the combustion wave proceeds in multiple stages with the formation of NiO and Ni(OH)_2_, which are reduced to Ni^0^. Higher ethylenediamine content in the complex leads to a higher content of metal in the solid products of combustion. However, different ratios of oxidized and reduced forms of nickel do not affect the initial activation temperature of nickel catalysts in the presence of CO_2_. It was noted that, after activation, the sample obtained from [Ni(C_2_H_8_N_2_)_2_](NO_3_)_2_ exhibited the highest activity in CO_2_ methanation. Thus, this complex is a promising precursor for CO_2_ methanation catalysts, and its synthesis requires only a small amount of ethylenediamine.

## 1. Introduction

In order to decrease the effect on the environment, new solvent-free methods need to be developed for the synthesis of catalysts, including CO_2_ methanation (or CO_2_ hydrogenation) catalysts. Currently, interest in this reaction is growing, since it allows a reduction in the carbon footprint of industry and energy while also solving the problem of the chemical storage of hydrogen by producing synthetic natural gas [1,2,3].

CO_2_ + 4H_2_ ↔ CH_4_ + 2H_2_O            ΔH_298K_ = −165 kJ/mol (1)

Traditionally, for this process, nickel catalysts are prepared using solvents. In this case, it is necessary to separate the catalyst from the solution and calcinate it at a high temperature to remove the solvent and form the active phase. These stages are common to most methods of CO_2_ methanation catalyst synthesis: co-precipitation [4,5], the sol–gel method [6] and incipient wetness impregnation [7,8]. In the method of solid-state combustion (SSC), the use of solvents and the heat treatment of catalysts can be avoided. For this reason, stable nickel complexes containing high-energy ligands and oxidizing anions were used as precursors of active components [9,10]. It is preferable to use nitrogen-rich organic compounds as ligands since their thermal decomposition is accompanied by ammonia evolution, which contributes to the reduction of nickel oxide formed during SSC [11].

3NiO + 2NH_3_ → 3Ni + 3H_2_O + N_2_ (2)

In addition, the metal–organic complexes should not contain crystallization water, the evaporation of which will cause heat loss in the high-temperature zone of the combustion wave.

Water-free nickel complexes can also be synthesized without the use of solvents, which will additionally increase the environmental friendliness of the catalyst preparation. In previous work, we have proposed the solvent-free synthesis of metal–organic nickel complexes by the addition of nickel nitrate or perchlorate crystal hydrate to melted imidazole at 90 °C. Within a few minutes, the solid product was formed, and its color changed from green to purple. The formation of [Ni(C_3_H_4_N_2_)_6_](NO_3_)_2_ and [Ni(C_3_H_4_N_2_)_6_](ClO_4_)_2_ complexes was confirmed by IR spectroscopy and powder XRD. Using these compounds, the bulk [12] and supported [13] nickel catalysts were prepared by SSC. The resulting solid combustion products consisted of both oxidized and reduced nickel phases. It was noted that the phase composition depends on the nature of the oxidizing anion. It should be noted that the authors of [14,15,16,17] discussed the effect of the nature of the nitrogen-containing ligand on the composition of the nickel-containing phases formed during the solution combustion synthesis. However, this method does not involve separating the complexes from the solution. The most studied approach is the formation of a metal-containing phase in the presence of glycine [18]. An increase in its content in the reaction medium leads to an increase in the proportion of reduced nickel [19,20,21]. This poses an obvious question: “How does the amount of high-energy ligand in the nickel complex affect the composition of nickel-containing phases formed during the SSC?” A similar important issue is the influence of the anion nature on the formation of nickel-containing phases during SSC.

In this work, nickel complexes containing two or three molecules of ethylenediamine and nitrate or perchlorate anions were synthesized without solvents. Their thermochemical properties and the phase compositions of their combustion products have been studied. The products of their solid-state combustion were tested as CO_2_ methanation catalysts, which were activated in the presence of CO_2_.

## 2. Materials and Methods

### 2.1. Materials

For the synthesis of the complexes, the following reagents were used: Ni(NO_3_)_2_·6H_2_O (GOST 4055-70, 98%); Ni(ClO_4_)_2_·6H_2_O (TU 6-09-02-118-86, 98%); and ethylenediamine (En) C_2_H_8_N_2_ (CAS 107-15-3, 99%).

### 2.2. Synthesis of Nickel Complexes

#### 2.2.1. Solvent-Free Synthesis

The synthesis was performed in a ceramic crucible at room temperature. In order to synthesize the nickel complexes, ethylenediamine (0.02 or 0.03 mol) was added to nickel (II) salt (0.01 mol) with stirring. The color of the reaction mixture changed from green to purple and then quickly crystallized as a powder. The obtained [Ni(C_2_H_8_N_2_)_2_](NO_3_)_2_, [Ni(C_2_H_8_N_2_)_3_](NO_3_)_2_ and [Ni(C_2_H_8_N_2_)_3_](ClO_4_)_2_ samples were dried under vacuum in a desiccator and stored over P_2_O_5_. The product yield was 95 ± 2%.

#### 2.2.2. Synthesis in a Solution

Ethylenediamine (0.04 or 0.08 mol) was added to a solution of 0.02 mol nickel salt in 10 mL of ethanol with vigorous stirring. The temperature of the solution increased, and the color changed from green to purple. The crystal precipitate was filtered and washed with a small amount of cold water and ethanol. It was dried in a vacuum and then stored in a desiccator over P_2_O_5_. The yields were 73, 92 and 96% for [Ni(C_2_H_8_N_2_)_2_](NO_3_)_2_, [Ni(C_2_H_8_N_2_)_3_](NO_3_)_2_ and [Ni(C_2_H_8_N_2_)_3_](ClO_4_)_2_, respectively.

### 2.3. Preparation of Samples by Solid-State Combustion of Nickel Complexes

The solid-state combustion procedure was performed in air. A corundum crucible with the complex was placed on the surface of a hot plate (IKA C-Mag HS 4, Königswinter, Germany) at a predetermined temperature of 500 °C. The sample was rapidly heated for several minutes, and spontaneous gasification was observed. The solid products of the complexes’ combustion were fine powders. They were stored in a desiccator over P_2_O_5_.

### 2.4. Characteristics of Nickel Complexes and Solid Products of Their Combustion

Inductively coupled plasma atomic emission spectrometry employing an Optima 4300 DV (PerkinElmer, Waltham, MA, USA) was performed to determine the Ni content. The carbon, hydrogen and nitrogen contents were analyzed with the use of an automatic CHNS analyzer (EURO EA 3000; Euro Vector S.p.A., Castellanza, Italy). The combustion of the complexes (0.5–2 mg) was performed at 1050 °C in a flow of O_2_/He. The measurement error was in the range of ±3%. The obtained results are given in Table 1.

The infrared spectra of [Ni(C_2_H_8_N_2_)_2_](NO_3_)_2_, [Ni(C_2_H_8_N_2_)_3_](NO_3_)_2_ and [Ni(C_2_H_8_N_2_)_3_](ClO_4_)_2_ were recorded using an Agilent Cary 600 spectrometer (Agilent Technologies, Santa Clara, CA, USA) with a Gladi ATR attachment (PIKE Technologies, Madison, WI, USA).

The powder X-ray diffraction (XRD) patterns of the synthesized complexes and the solid products of their combustion were recorded in a 2θ range from 5 to 70° with a step of 0.05° (accumulation time—2 s) using a D8 Advance diffractometer with a Lynxeye linear detector (Bruker AXS GmbH, Karlsruhe, Germany). CuK_α_ radiation (λ = 1.5418 Å) was used. The composition of the solid products was identified by the Rietveld method [22] using the TOPAS program (TOPAS—Total Pattern Analysis System, Version 4.2, Bruker AXS). Metallic silicon was used as a reference material for instrumental broadening. The size of the coherent scattering region (CSR) was calculated using LVol-IB values (LVol-IB—the volume-weighted mean column height based on integral breadth) and LVol-FWHM (the volume-weighted mean column height based on full width at half maximum, k = 0.89).

Crystallographic data (excluding structure factors) for the nickel complexes were obtained from the Cambridge Crystallographic Data Centre (CCDC, 12 Union Road, Cambridge CB21EZ, UK, http://www.ccdc.cam.ac.uk, 20 February 2023). Copies of the data can be obtained free of charge upon quoting the following depository numbers: CCDC-262247 for [Ni(C_2_H_8_N_2_)_2_](NO_3_)_2_, CCDC-1268271 for [Ni(C_2_H_8_N_2_)_3_](NO_3_)_2_ and CCDC-1320608 for [Ni(C_2_H_8_N_2_)_3_](ClO_4_)_2_·H_2_O. Theoretical powder patterns for [Ni(C_2_H_8_N_2_)_2_](NO_3_)_2_, [Ni(C_2_H_8_N_2_)_3_](NO_3_)_2_ and [Ni(C_2_H_8_N_2_)_3_](ClO_4_)_2_·H_2_O were plotted from the CIF available in [23,24,25], respectively.

### 2.5. Study of Thermochemical Properties

The thermochemical properties of the prepared complexes (5 mg) were studied on a Netzsch STA 449 C Jupiter instrument equipped with a DSC/TG holder (NETZSCH, Selb, Germany). The samples were heated from room temperature to 500 °C at a rate of 5 °C·min^−1^ in helium. The DSC curves were obtained at heating rates of 2.5, 5, 10 and 15 °C·min^−1^.

The kinetic parameters of the thermochemical transformations were calculated by inverse modeling using a genetic algorithm (GA), which has been successfully used in recent years [26,27,28,29,30]. The modeling was carried out employing the Coats–Redfern equation [31]:

(3) $$g\left(\alpha \right)=\frac{ART^{2}}{\beta E}\left(1-\frac{2RT}{E}\right)\exp\left(-\frac{E}{RT}\right)$$

where α is the extent of the reaction; g(α) is the integral form of the reaction model; A is the preexponential factor; E is the activation energy; R is the gas constant; T is temperature; and β is the heating rate.

Taking into account that usually $1-\frac{2RT}{E}≅1$, its linear approximation is widely used in practice:

(4) $$\ln\left(\frac{g(\alpha )}{T^{2}}\right)=\ln\left(\frac{AR}{\beta E}\right)-\frac{E}{RT}$$

The GA was chosen as the objective function:

(5) $$F=\frac{1}{N}\sum_{i}^{N}\left({\left(\alpha_{i}^{theor}{-\alpha }_{i}^{exp}\right)}^{2}+{\left({\frac{{d\alpha }^{theor}}{dt}}_{i}-{\frac{{d\alpha }^{exp}}{dt}}_{i}\right)}^{2}\right)$$

where N is the number of experimental points; t is time; $\alpha_{i}^{theor}$ and $\alpha_{i}^{exp}$ are simulated and experimental conversion fractions at temperature i; and ${\frac{{d\alpha }^{theor}}{dt}}_{i}$ and ${\frac{{d\alpha }^{exp}}{dt}}_{i}$ are simulated and experimental reaction rates at temperature i [26].

The inverse modeling of the processes was carried out by employing the unconstrained variant of the GA; the initial population range for lgA was from 10 to 30, and that for Ea was from 200 to 400 kJ/mol. Each run of the hybrid GA was repeated 4 times to ensure that the acquired solutions were repeatable.

To compare the thermal stability of the nickel complexes, the critical temperature of the thermal explosion (T_b_) was calculated based on the DSC data at several heating rates (β):

(6) $$T_{b}=T_{0}+\frac{\partial T_{e}}{\partial \left(ln\beta \right)}$$

where T_e_ is the onset temperature as a function of β; T_0_ is the onset temperature extrapolated to β = 0 °C·min^−1^ using the 3rd-order polynomial fit [32].

### 2.6. CO_2_ Methanation Procedure

A fixed-bed reactor with an inner diameter of 10 mm was used to carry out catalytic experiments under atmospheric pressure. The catalyst mass was 0.3 g. A tubular furnace was used to heat the reaction mixture. The temperature was controlled by a thermocouple installed in the catalyst bed. Nickel catalysts were not reduced before the experiment. Catalytic tests were carried out at various temperatures from 150 to 350 °C with a step of 50 °C.

A reaction mixture diluted with Ar (H_2_:CO_2_:Ar = 16:4:80) was fed into the reactor at a gas mass space velocity of 19,000 mL·g_cat_^−1^·h^−1^. The reactant feed rate was chosen in such a way as to exclude the condensation of the formed water at the outlet of the reactor and on the walls of the gas cell of the FTIR spectrometer. The flow rates were controlled using an RRG-12-36 flow meter (“Eltochpribor”, Moscow, Russia) with an accuracy of 1%. The gas composition at the outlet of the reactor was monitored using an Agilent Cary 630 FTIR spectrometer (Agilent Technologies Australia, Melbourne, Australia) with an internal volume of the gas cell of 100 cm^3^ and an optical path length of 10 cm.

## 3. Results and Discussion

### 3.1. Solvent-Free Synthesis of the Nickel Complexes with Ethylenediamine

**Figure 1:**
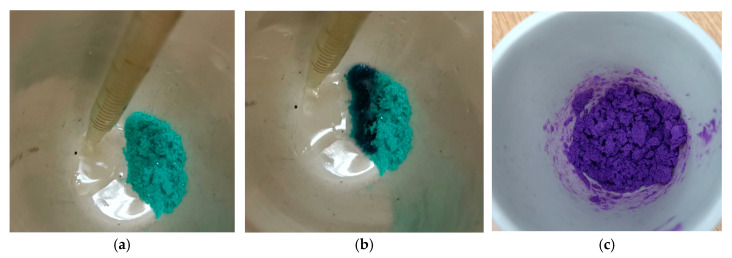
Synthesis of tris(ethylenediamine)nickel(II) nitrate without solvent: (**a**) initial state; (**b**) 0.5 min; (**c**) 2 min.

Bis(ethylenediamine)nickel(II) nitrate [Ni(C_2_H_8_N_2_)_2_](NO_3_)_2_, tris(ethylenediamine)nickel(II) nitrate [Ni(C_2_H_8_N_2_)_3_](NO_3_)_2_ and tris(ethylenediamine)nickel(II) perchlorate [Ni(C_2_H_8_N_2_)_3_](ClO_4_)_2_ were chosen as the precursors of the CO_2_ methanation catalysts. The complexes were prepared by adding liquid ethylenediamine to the nickel salts at room temperature (Figure 1). The reaction mixture heated up to 90 °C during the synthesis due to the exothermic effect of the interaction between its components. This contributed to the evaporation of water released in the process. It should be noted that the elemental composition of the synthesized complexes is in agreement with the calculated values for [Ni(C_2_H_8_N_2_)_2_](NO_3_)_2_ and [Ni(C_2_H_8_N_2_)_3_](NO_3_)_2_ (Table 1). In the case of [Ni(C_2_H_8_N_2_)_3_](ClO_4_)_2_, the hydrogen and oxygen contents are higher than the calculated values, which may be due to the presence of water in this complex.

### 3.2. Study of the Nickel Complexes Synthesized without Solvent

**Figure 2:**
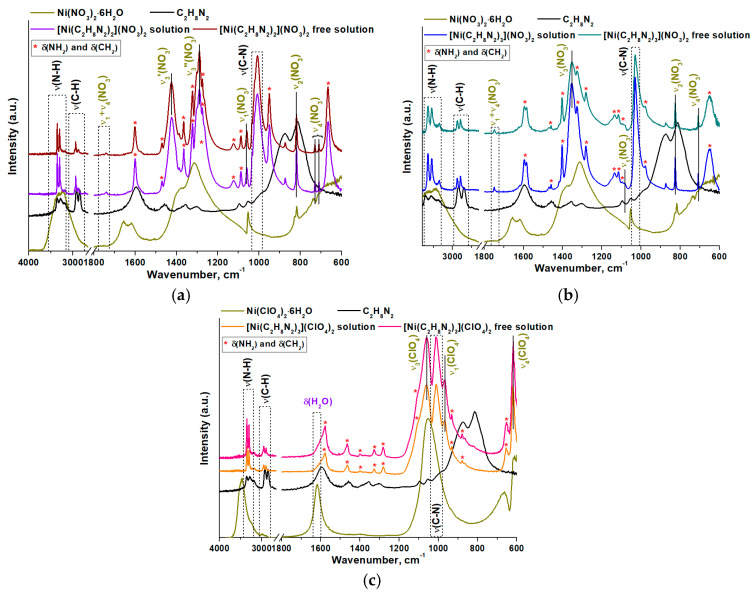
ATR FTIR data for obtained (**a**) [Ni(C_2_H_8_N_2_)_2_](NO_3_)_2_, (**b**) [Ni(C_2_H_8_N_2_)_3_](NO_3_)_2_ and (**c**) [Ni(C_2_H_8_N_2_)_3_](ClO_4_)_2_ complexes.

The ATR FTIR data (Figure 2) confirm that the [Ni(C_2_H_8_N_2_)_2_](NO_3_)_2_ and [Ni(C_2_H_8_N_2_)_3_](NO_3_)_2_ complexes do not contain water, since there are no absorption bands (a.b.) related to the stretching vibrations of the O–H group (3600–3400 cm^−1^) and deformation vibrations of water (1620 cm^−1^). Moreover, their FTIR spectra are identical to those of the complexes synthesized in ethanol.

An analysis of the FTIR spectra of the perchlorate complex showed that this compound is characterized by a shoulder in the high-frequency region of the absorption band with a clearly pronounced maximum at 1580 cm^–1^. The absorption at 1615 cm^−1^ can be attributed to the presence of water in this complex, which is consistent with elemental analysis data.

**Figure 3:**
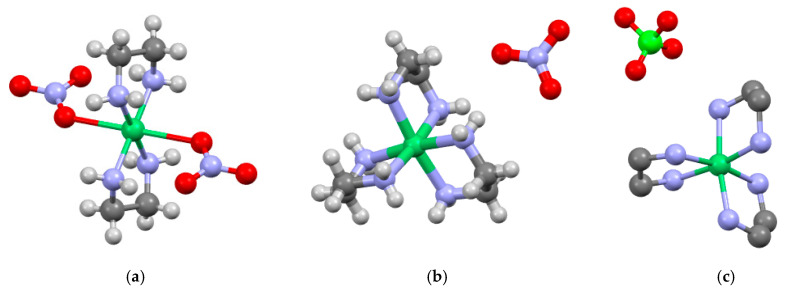
Structure of (**a**) [Ni(C_2_H_8_N_2_)_2_](NO_3_)_2_, (**b**) [Ni(C_2_H_8_N_2_)_3_](NO_3_)_2_ and (**c**) [Ni(C_2_H_8_N_2_)_3_](ClO_4_)_2_ (without hydrogen) complexes: green—Ni^2+^ ion; lilac—nitrogen atom; dark gray—carbon atom; light gray—hydrogen atom; red—oxygen atom.

Depending on the selected ratio of nickel and ethylenediamine, chelate coordination sites of NiN_4_ and NiN_6_ are formed during their interaction. Both nitrogen atoms of the ethylenediamine ligand form coordination bonds with the nickel ion, forming two or three five-membered heteroaromatic rings (Figure 3). However, in the [Ni(C_2_H_8_N_2_)_2_](NO_3_)_2_ complex, the coordination number of the nickel(II) ion is also 6 because of its direct interaction with nitrate anions (Figure 3a). The symmetry of the nitrate anion decreases and the vibrations of the atoms in the anion become nonequivalent.

The free nitrate anion is characterized by D_3h_ symmetry, which has four vibrational modes. The infrared-active modes are ν_2_—out-of-plane rocking (830 cm^−1^); ν_3_—antisymmetric N–O stretches (1350 cm^−1^); and ν_4_—in-plane deformations (710 cm^−1^) [33]. The ν_3_ and ν_4_ vibrations are doubly degenerate: i.e., they might split upon symmetry lowering. Indeed, in the FTIR spectrum of the [Ni(C_2_H_8_N_2_)_2_](NO_3_)_2_ complex, two a.b. at 710 and 728 cm^−1^ are observed in the region of the ν_4_ mode. Additionally, two a.b. at 1424 and 1289 cm^−1^ are present in the region of the ν_3_ mode against the background of deformation vibrations of NH_2_ and CH_2_ groups [34,35]. The splitting value is 135 cm^−1^, which corresponds to the monodentate coordination of the nitrate anion [36]. It was noted that in the FTIR spectrum of the [Ni(C_2_H_8_N_2_)_3_](NO_3_)_2_ complex, there are no additional a.b. for the ν_3_ and ν_4_ modes. Therefore, the symmetry of the nitrate anion is preserved when this complex is formed. Thus, it is located in the outer coordination sphere of the complex, as shown in Figure 3b.

When the symmetry of the perchlorate anion is lowered, the a.b. corresponding to ν_3_ (1100–1050 cm^−1^) and ν_4_ (640–610 cm^−1^) vibrations are also split [37]. However, in the IR spectrum of the [Ni(C_2_H_8_N_2_)_3_](ClO_4_)_2_ complex, the additional a.b. in the region of the ν_3_ and ν_4_ vibrational modes are absent. This indicates that the symmetry of the perchlorate anion is retained in the synthesized complex (Figure 3c).

**Figure 4:**
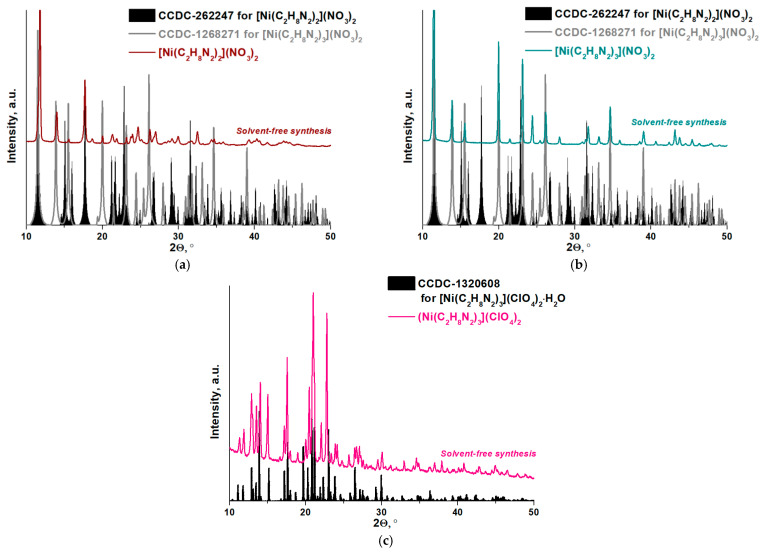
The power XRD data obtained for (**a**) [Ni(C_2_H_8_N_2_)_2_](NO_3_)_2_, (**b**) [Ni(C_2_H_8_N_2_)_3_](NO_3_)_2_ and (**c**) [Ni(C_2_H_8_N_2_)_3_](ClO_4_)_2_ complexes.

To identify the structures of the complexes synthesized without the solvents, [Ni(C_2_H_8_N_2_)_2_](NO_3_)_2_, [Ni(C_2_H_8_N_2_)_3_](NO_3_)_2_ and [Ni(C_2_H_8_N_2_)_3_](ClO_4_)_2_ were studied by powder XRD (Figure 4). It was found that all peaks in the XRD pattern of the complex containing the chelate coordination site of NiN_6_ match the theoretical powder pattern of [Ni(C_2_H_8_N_2_)_3_](NO_3_)_2_ plotted from the CIF available in [24].

The XRD pattern acquired for the complex with the chelate coordination site of NiN_4_ contains more peaks than the theoretical powder pattern of [Ni(C_2_H_8_N_2_)_2_](NO_3_)_2_ plotted from the CIF available in [23]. The most intense peaks can be attributed to synthesized bis(ethylenediamine)nickel(II) nitrate. The position of low-intensity peaks corresponds to the peaks characteristic of the complex with the chelate coordination site of NiN_6_. Therefore, [Ni(C_2_H_8_N)_2_](NO_3_)_2_ contains an insignificant impurity of [Ni(C_2_H_8_N_2_)_3_](NO_3_)_2_.

An analysis of the XRD pattern for the perchlorate complex showed that most of the peaks matched the theoretical powder pattern of [Ni(C_2_H_8_N_2_)_3_](ClO_4_)_2_·H_2_O plotted from the CIF available in [25]. However, small shifts of some peaks are observed. This can be explained by imperfections in the structure of the complex, which was not thermally treated.

Summing up the study of nickel complexes with ethylenediamine synthesized without solvents, it can be concluded that the addition of ethylenediamine to nickel nitrate and perchlorate hydrate causes the formation of [Ni(C_2_H_8_N_2_)_3_](NO_3_)_2_, [Ni(C_2_H_8_N_2_)_2_](NO_3_)_2_ and [Ni(C_2_H_8_N_2_)_3_](ClO_4_)_2_·H_2_O complexes. Their composition and structure depend on the stoichiometric ratio of nickel to ethylenediamine in the reaction mixture. In addition, this method allows the partial or complete removal of water from the nickel complexes without additional heating.

### 3.3. Thermochemical Properties of the Nickel Complexes with Ethylenediamine

**Figure 5:**
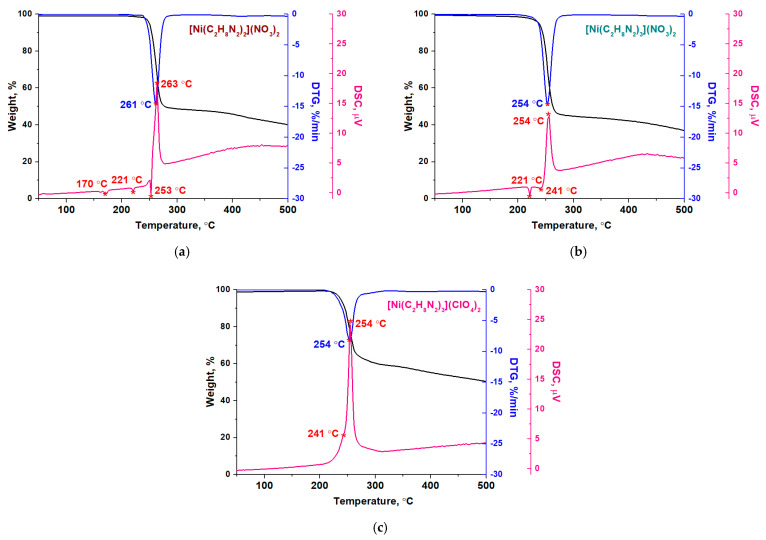
The thermal analysis data obtained for (**a**) [Ni(C_2_H_8_N_2_)_2_](NO_3_)_2_, (**b**) [Ni(C_2_H_8_N_2_)_3_](NO_3_)_2_ and (**c**) [Ni(C_2_H_8_N_2_)_3_](ClO_4_)_2_ complexes (5 mg, helium, 20 mL·min^−1^, 5 °C·min^−1^).

The thermochemical transformations of [Ni(C_2_H_8_N_2_)_3_](NO_3_)_2_, [Ni(C_2_H_8_N_2_)_2_](NO_3_)_2_ and [Ni(C_2_H_8_N_2_)_3_](ClO_4_)_2_·H_2_O complexes were studied (Figure 5).

According to the thermal analysis data (Figure 5), the synthesized complexes begin to decompose at temperatures above 200 °C and reach the maximum mass loss rate at 257 ± 4 °C. In addition, the DTG curves do not contain maxima related to the thermal decomposition of nickel nitrate [38,39] or nickel perchlorate [40] and ethylenediamine, which boils at 117 °C [41]. Thus, the starting compounds reacted and became a part of the resulting complexes.

Taking only the mass loss of nickel complexes during heating into account, it seems that their thermal decomposition is a one-stage process. However, the DSC curves (Figure 5, dark pink line) show that complicated thermochemical transformations of the complexes occur. The endothermic effects at temperatures below 240 °C can be attributed to phase transformations, since they are not accompanied by a change in the mass of the samples. It should be noted that when the [Ni(C_2_H_8_N_2_)_3_](NO_3_)_2_ complex is heated, only one endothermic effect is observed at 221 °C (Figure 5b). A similar effect on the DSC curve (but much less pronounced) is also characteristic of the [Ni(C_2_H_8_N_2_)_2_](NO_3_)_2_ complex (Figure 5a). Therefore, it can be assumed that this complex contains an impurity of the complex with three molecules of ethylenediamine, as noted earlier according to XRD data (Figure 4a).

In the temperature range above 240 °C, there is a superposition of endothermic and exothermic effects for complexes with nitrate anions, especially for a NiN_4_ coordination site. The reason for this effect may be the simultaneous removal of ethylenediamine from the coordination sphere of nickel (endothermic peak) and its rapid oxidation by nitrate anions (exothermic peak), as described in [42,43]. A weak endo-effect at 241 °C (Figure 5c) is also observed in the case of the complex with perchlorate anions.

To identify the thermal decomposition stages of the nickel complexes and calculate their kinetic parameters, an analysis of the DTG data was carried out using the inverse modeling method with a genetic algorithm [26,27,28,29]. Both one-stage and two-stage models of the thermal decomposition of the complexes were created, but it was not possible to satisfactorily describe the decomposition of [Ni(C_2_H_8_N_2_)_2_](NO_3_)_2_ in two stages using the genetic algorithm. The determination of the kinetic parameters of the decomposition of the complexes in one stage was based on the Mampel kinetic model (Table S1, Figure S1a). In the case of the two-stage simulation, different kinetic models were tried (Table S1, Figure S1b,c). The choice of the most suitable model was determined by the following criteria [43,44,45,46]: the first stage should describe the removal of ethylenediamine (weight loss of 17 ± 5%), and the value of the activation energy for this stage should be acceptable (about 200 kJ·mol^−1^). The calculated kinetic parameters are presented in Table 2.

The obtained results indicate that there are differences in the mechanisms of the thermal decomposition of the nickel complexes. In the case of the [Ni(C_2_H_8_N_2_)_2_](NO_3_)_2_ complex, the highest activation energy is observed, which confirms its higher thermal stability compared to complexes with three ligands (Table 2). In addition, the stage of the ethylenediamine release could not be separated. Consequently, when this complex is heated, only the oxidation of the ligand by nitrate anions takes place.

At the same time, the thermal decomposition of [Ni(C_2_H_8_N_2_)_3_](NO_3_)_2_ and [Ni(C_2_H_8_N_2_)_3_](ClO_4_)_2_ is characterized by two stages (Figure S1b,c). The first stage is the removal of one ligand by the contracting cylinder mechanism with an activation energy of 192 ± 1 kJ/mol. However, the sample mass loss is greater than the calculated mass loss from the removal of the ethylenediamine molecule. This difference is small (~2%) but indicates the implementation of a parallel process. Presumably, the pyrolysis of ethylenediamine or its oxidation occurs. In the second stage, the remaining ethylenediamine is mainly oxidized by nitrate anions. It was noted that the resulting solid product (Figure 5) had a greater mass (40–50%) than would be expected (17–25%). The reason may be the presence of products of the incomplete decomposition of complexes due to their thermal stability.

The critical temperature of the thermal explosion characterizes the thermal stability of materials. In this work, it was calculated for [Ni(C_2_H_8_N_2_)_2_](NO_3_)_2_, [Ni(C_2_H_8_N_2_)_3_](NO_3_)_2_ and [Ni(C_2_H_8_N_2_)_3_](ClO_4_)_2_ using the method described by Xue et al. [32]. To determine these values, DSC data at various heating rates (Figure S2) were analyzed. The obtained results are given in Table 3.

Table 3 shows that the nickel perchlorate complex with three ethylenediamine molecules is the least stable. It is noted that a decrease in the amount of ethylenediamine in the nickel nitrate complex leads to an increase in the temperature of its thermal degradation. Apparently, the removal of one molecule of the ligand destabilizes the [Ni(C_2_H_8_N_2_)_3_](NO_3_)_2_ complex and accelerates the oxidation of the remaining ligands by anions. In addition, the direct contact of the nitrate anions with the nickel cation in [Ni(C_2_H_8_N_2_)_2_](NO_3_)_2_ is of great importance (Figure 3).

Summarizing the results of studying the thermal decomposition of the synthesized nickel complexes with ethylenediamine (Table 1), it was concluded that they are thermally stable up to 200 °C. With a further increase in temperature, they decompose in one or two steps, which are determined by the amount of ligand in the nearest coordination sphere of nickel. These stages were separated using the inverse modeling method with the genetic algorithm.

### 3.4. Study of the Solid Products of the Gasification of Nickel Complexes

**Figure 6:**
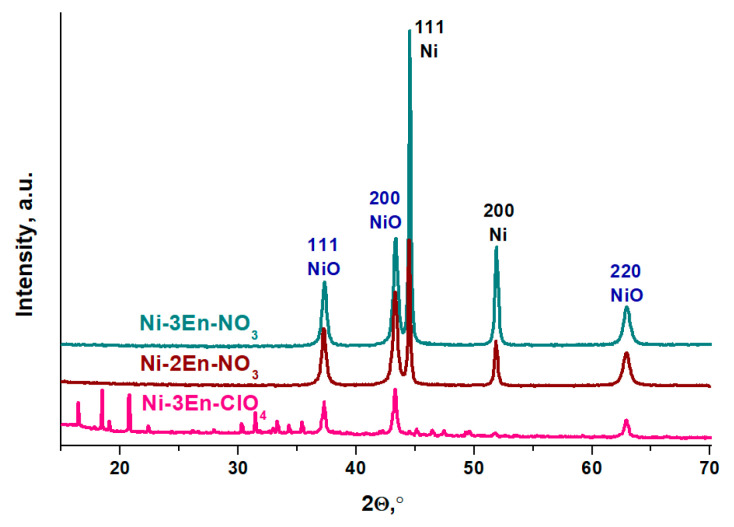
The XRD patterns of Ni-2En-NO_3_, Ni-3En-NO_3_ and Ni-3En-ClO_4_ samples.

The synthesized nickel complexes with ethylenediamine were used as precursors for the synthesis of CO_2_ methanation catalysts by SSC. During the procedure, the formation of solid products containing nickel was observed. The samples obtained from the [Ni(C_2_H_8_N_2_)_2_](NO_3_)_2_, [Ni(C_2_H_8_N_2_)_3_](NO_3_)_2_ and [Ni(C_2_H_8_N_2_)_3_](ClO_4_)_2_ complexes were designated as **Ni-2En-NO_3_**, **Ni-3En-NO_3_** and **Ni-3En-ClO_4_**, respectively. Their phase compositions were studied by XRD (Figure 6).

According to the XRD data (Figure 6), the Ni-2En-NO_3_ and Ni-3En-NO_3_ samples contain two phases: metallic nickel (PDF 04-0850) and its oxide—NiO (PDF 47-1049). In the process of the gasification of the perchlorate complex, nickel oxide is only formed since the perchlorate anion is a stronger oxidizing agent than nitrate. However, this sample has impurities with a crystal lattice, which cannot be unambiguously attributed to the original complex (Figure 4c), nickel perchlorate [47], nickel carbide [48], nickel nitride [49] or nickel chloride [50]. It can be assumed that the impurities are carbonization products of the organic ligand. However, they cannot be attributed to graphite materials since there is no peak attributable to the interplanar packing of aromatic systems, which is indexed as the (002) peak [51]. It is more likely that the impurities are a multicomponent mixture containing amorphous carbon nitride since the IR spectra (Figure S3) contain absorption bands characteristic of this compound [52].

Using XRD data, the contents of each nickel phase in the Ni-2En-NO_3_, Ni-3En-NO_3_ and Ni-3En-ClO_4_ samples were calculated. It was also noted that the composition of the samples is dominated by nickel oxide (Table 4), which is formed during the decomposition of the complexes.

Metallic nickel is contained in samples obtained from nitrate complexes, and the more molecules of ethylenediamine in the nearest coordination sphere of nickel, the more metal phase there is. It is known from the literature [53,54,55,56] that the decomposition of nitrogen-containing organic compounds produces ammonia, which facilitates the reduction of the metal (Equation (2)). Indeed, according to mass spectrometry data (Figure S4), ammonia is one of the products generated during the rapid heating of nickel nitrate complexes with ethylenediamine.

Taking into account the size of the coherent scattering region (CSR), the sizes of crystallites were calculated for each phase of Ni-2En-NO_3_, Ni-3En-NO_3_ and Ni-3En-ClO_4_. It was found that the obtained values do not exceed 60 nm (Table 4). Therefore, intense gas evolution leads to the high dispersion of the metal-containing phase. In the case of nitrate complexes, oxidized and reduced forms of metals with similar crystallite sizes are formed. Nickel oxide nanoparticles obtained from the nickel perchlorate complex are characterized by larger crystallite sizes. Thus, it can be concluded that the phase composition of the solid products obtained from nickel complexes with ethylenediamine depends on the number of ligands in the nearest coordination sphere of the metal and the nature of the anion. Of particular interest is the presence of a metal phase in the samples obtained by SSC, since it can enable the formation of the active phase of the catalyst without the reduction step.

### 3.5. CO_2_ Methanation: Activation of the Nickel Catalysts Synthesized by SSC from the Nickel Complexes with Ethylenediamine

**Figure 7:**
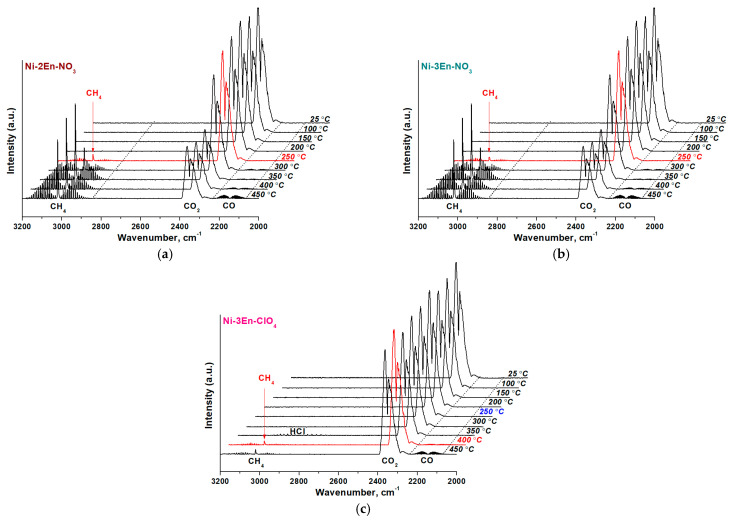
FTIR spectra of the gas mixture at the reactor outlet during the (**a**) Ni-2En-NO_3_, (**b**) Ni-3En-NO_3_ and (**c**) Ni-3En-ClO_4_ catalysts’ activation at different temperatures.

Nickel catalysts are widely used for CO_2_ and CO methanation in the purification of hydrogen from carbon oxides due to their low cost [57,58]. Typically, to form a metal phase, the activation of nickel catalysts is carried out in a hydrogen flow at high temperatures [59]. In this work, this procedure was performed in the flow of the reaction mixture (H_2_:CO_2_:Ar = 16:4:80), which is more practical. The presence of methane in the reaction medium at the outlet of the reactor indicated the formation of a catalytically active phase, which accelerated CO_2_ methanation. The gas composition was determined using FTIR spectroscopy (Figure 7).

According to the results obtained (Figure 7), in the reaction medium, methane begins to be fixed when the Ni-2En-NO_3_ and Ni-3En-NO_3_ catalysts are heated at 250 °C. Here, the intensity of methane absorption bands is higher for the Ni-2En-NO_3_ sample. In the case of the sample obtained from the nickel perchlorate complex with ethylenediamine, methane formation is observed only when the temperature reaches 400 °C (Figure 7c). It should be noted that this is preceded by the release of hydrogen chloride at 350 °C, which is the dominant product of the complex decomposition (Figure S4c). Thus, the nickel is simultaneously reduced with the removal of products of the incomplete decomposition of the complexes, as noted above (Figure S3).

To explain the differences in catalyst activation in the reaction medium, they were studied before and after the reaction by XPS. From the XPS survey spectra (Figure S5), it can be seen that nickel, oxygen, carbon, chlorine and nitrogen are present on the surface of the initial samples. Here, the solid product from the nickel perchlorate complex contains a significant amount of chlorine and nitrogen, as well as 2 times less nickel (Table 5). This once again proves the presence of products of its incomplete decomposition in Ni-3En-ClO_4_.

**Figure 8:**
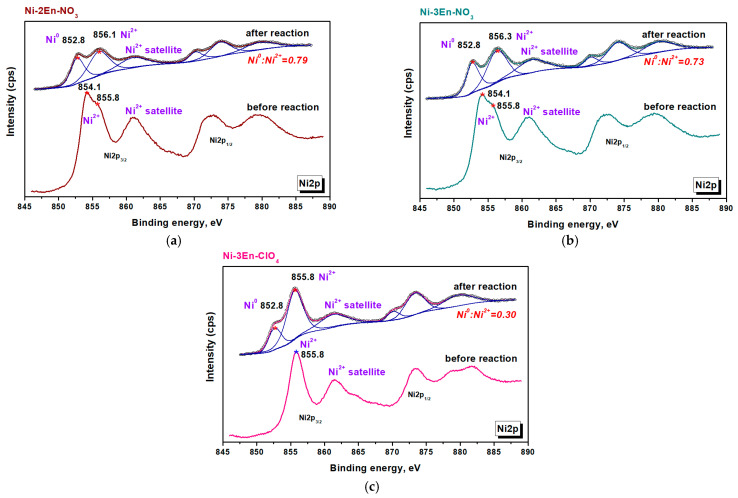
XPS spectra of Ni2p region for (**a**) Ni-2En-NO_3_, (**b**) Ni-3En-NO_3_ and (**c**) Ni-3En-ClO_4_ samples before and after activation at 450 °C in the reaction medium (H_2_:CO_2_:Ar = 16:4:80).

A detailed analysis of the XPS spectra (Figure 8) was performed to identify the electronic state of nickel. It was found that oxidized nickel was present on the surface of samples only before activation. The XPS spectra of the Ni-2En-NO_3_ and Ni-3En-NO_3_ samples in the Ni2p3/2 region show peaks at ~854.2 and ~855.8 eV, in agreement with the binding energies of nickel oxide and hydroxide, respectively [60]. The Ni2p3/2 XPS spectrum of Ni-3En-ClO_4_ can be deconvoluted into one peak with a binding energy of ~855.8 eV. Therefore, only nickel hydroxide is present on the surface of this sample, which probably covers the nickel oxide detected by XRD (Table 4).

After the activation of Ni-2En-NO_3_, Ni-3En-NO_3_ and Ni-3En-ClO_4_ samples at 450 °C, an additional maximum is observed at 852.8 eV in the Ni2p3/2 region, corresponding to the reduced metal [61]. The amount of metal in the reduced form depends on the composition of the complex. Thus, under reaction conditions, more than 70% of nickel is reduced in samples obtained from complexes with nitrate anions, and only 30% of the reduced metal contains the Ni-3En-ClO_4_ catalyst after activation. Such a low degree of nickel reduction may be due to the presence of nickel hydroxide in the sample, which has a higher reduction temperature than nickel oxide [62]:

NiO + H_2_ → Ni + H_2_O            250–400 °C (7)

Ni(OH)_2_ + H_2_ → Ni + 2H_2_O            350–600 °C (8)

Thus, Ni-2En-NO_3_ and Ni-3En-NO_3_ catalysts more effectively accelerate CO_2_ conversion since the active phase is formed from nickel oxide, which begins to be reduced at 250 °C (Figure 7). This phase predominates in the Ni-2En-NO_3_ sample (Table 4), which is characterized by more intense absorption in the spectral region of methane (Figure 7a).

Another important result of this research is the conclusion that the metal formed during the complexes’ combustion (Table 4) does not affect CO_2_ methanation. Apparently, metal particles are located in the bulk of the sample (due to attraction to each other under the action of their own magnetic field) and covered with an oxygen-containing nickel phase.

## 4. Conclusions

This work is a comprehensive study, including (I) the solvent-free synthesis of nickel complexes as catalyst precursors and (II) the study of their thermochemical properties that affect (III) the formation of the active phase (IV) catalyzing CO_2_ methanation after activation. Energy-rich complexes with two and three molecules of ethylenediamine in the nearest coordination sphere of nickel were chosen as initial compounds. The counterions of nickel were nitrate and perchlorate anions that acted as oxidizing agents.

(I) The complexes were synthesized by mixing nickel nitrate hexahydrate or nickel perchlorate hexahydrate with ethylenediamine without the use of a solvent, which excludes their separation from the solvent and drying. The structure and the phase composition of the prepared complexes were confirmed by IR spectroscopy and powder XRD. As a result, [Ni(C_2_H_8_N_2_)_2_](NO_3_)_2_, [Ni(C_2_H_8_N_2_)_3_](NO_3_)_2_ and [Ni(C_2_H_8_N_2_)_3_](ClO_4_)_2_ complexes were obtained, whose composition was determined by the ratio of ethylenediamine and nickel salt. However, the addition of a third ethylenediamine molecule to the chelate coordination site of NiN_4_ could not be completely avoided, and the [Ni(C_2_H_8_N_2_)_2_](NO_3_)_2_ complex contains an insignificant impurity of [Ni(C_2_H_8_N_2_)_3_](NO_3_)_2_.
(II) To understand the processes that take place during the formation of the nickel-containing phase in the combustion wave, the thermochemical transformations of the synthesized complexes were studied. It was found that they are thermally stable up to 230 °C and then decompose with a weight loss of almost 60%. The kinetic analysis of the thermal decomposition of the synthesized complexes and the calculation of their kinetic parameters were carried out by inverse modeling with a genetic algorithm. It was determined that the thermal decomposition of complexes with three ethylenediamine molecules has two stages: (1) the removal of one ligand molecule, and (2) the oxidation of the ligand by anions. In the case of the [Ni(C_2_H_8_N_2_)_2_](NO_3_)_2_ complex, thermal decomposition is described by only one stage. In addition, this complex has the highest activation energy of thermolysis and the highest critical temperature of thermal explosion, which indicates the high thermal stability of the chelate site of NiN_4_.
(III) The synthesized nickel complexes with ethylenediamine were used as precursors for the synthesis of CO_2_ methanation catalysts by SSC. It was shown by powder XRD and XPS that the obtained samples consist of oxidized (NiO, Ni(OH)_2_) and reduced (Ni^0^) forms of nickel. It was noted that the fewer the ethylenediamine molecules in the nickel coordination sphere of nitrate complexes, the more nickel oxide contained in the combustion products.
(IV) The activation of combustion products of the [Ni(C_2_H_8_N_2_)_2_](NO_3_)_2_, [Ni(C_2_H_8_N_2_)_3_](NO_3_)_2_ and [Ni(C_2_H_8_N_2_)_3_](ClO_4_)_2_ complexes in the reaction medium (H_2_:CO_2_:Ar = 16:4:80) of CO_2_ methanation was studied. It was found that in the presence of Ni-2En-NO_3_ and Ni-3En-NO_3_, methane already begins to form at 250 °C, which is 100 °C lower than the activation temperature of an industrial NIAP-07-01 catalyst (Figure S6). At an even higher temperature (400 °C), an active phase is formed in the sample obtained from the nickel perchlorate complex. The comparison of previously obtained experimental data [12,13] with the results of this work shows that the Ni-2En-NO_3_ sample with a high content of nickel oxide showed the highest activity in CO_2_ methanation (Figure S7). Thus, the [Ni(C_2_H_8_N_2_)_2_](NO_3_)_2_ complex is a promising precursor for the CO_2_ methanation catalyst, especially since less ethylenediamine is required for its solvent-free synthesis.

## Figures and Tables

**Table 1:** Characteristics of the synthesized nickel complexes.

Complex, Molar Mass	Composition, %		Gross Formula
	Calculated	Found	
[Ni(C_2_H_8_N_2_)_2_](NO_3_)_2_ NiC_4_H_16_N_6_O_6_ 303 g·mol^−1^	Ni—19.5 C—15.8 H—5.3 N—27.7 O—31.7	Ni—19.4 C—16.3 H—5.9 N—29.0 Other (“O”) ^1^—29.4	NiC_4.1_H_17.9_N_6.3_“O”_5.6_
[Ni(C_2_H_8_N_2_)_3_](NO_3_)_2_ NiC_6_H_24_N_8_O_6_ 363 g·mol^−1^	Ni—16.3 C—19.8 H—6.6 N—30.9 O—26.4	Ni—16.8 C—19.6 H—7.3 N—31.2 Other (“O”) ^1^—25.1	NiC_5.7_H_25.6_N_7.8_“O”_5.5_
[Ni(C_2_H_8_N_2_)_3_](ClO_4_)_2_ NiC_6_H_24_N_6_Cl_2_O_8_ 438 g·mol^−1^	Ni—13.5 C—16.4 H—5.5 N—19.2 Cl—16.2 O—29.2	Ni—12.0 C—16.5 H—5.8 N—19.5 Cl—15.7 Other (“O”) ^1^—30.5	NiC_6.8_H_28.5_N_6.8_Cl_2.2_“O”_9.4_ or NiC_6.8_H_26.5_N_6.8_Cl_2.2_“O”_8.4_·H_2_O

^1^ Supposedly oxygen.

**Table 2:** Results for the modeling of the thermal decomposition of [Ni(C_2_H_8_N_2_)_2_](NO_3_)_2_, [Ni(C_2_H_8_N_2_)_3_](NO_3_)_2_ and [Ni(C_2_H_8_N_2_)_3_](ClO_4_)_2_ complexes (5 mg, helium, 20 mL·min^−1^, 5 °C·min^−1^).

Complex	Temperature Range, °C	Stage	R^2^	Kinetic Model ^1^	lgA	E, kJ·mol^−1^	Mass Loss, wt%
[Ni(C_2_H_8_N_2_)_2_](NO_3_)_2_	230–300	1	0.9986	1	35.1	378	51
[Ni(C_2_H_8_N_2_)_3_](NO_3_)_2_	200–300	1	0.9992	1	29.6	319	54
		2	0.9988	5	16.6	193	18
				1	33.1	355	36
[Ni(C_2_H_8_N_2_)_3_](ClO_4_)_2_	205–270	1	0.9997	1	23.8	261	36
		2	0.9998	5	16.6	191	16
				1	32.8	351	20

^1^ Table S1.

**Table 3:** The onset temperature and the critical temperature of the thermal explosion for [Ni(C_2_H_8_N_2_)_2_](NO_3_)_2_, [Ni(C_2_H_8_N_2_)_3_](NO_3_)_2_ and [Ni(C_2_H_8_N_2_)_3_](ClO_4_)_2_ complexes (5 mg, helium, 20 mL·min^−1^).

Complex	β_i_, °C·min^−1^	T_e_, °C	T_0_, °C	$\frac{\partial T_{e}}{\partial \left(ln\beta \right)}$, °C	T_b_, °C
[Ni(C_2_H_8_N_2_)_2_](NO_3_)_2_	2.5	252	246	11	257
	5	258			
	10	269			
	15	271			
[Ni(C_2_H_8_N_2_)_3_](NO_3_)_2_	2.5	239	218	15	233
	5	252			
	10	260			
	15	265			
[Ni(C_2_H_8_N_2_)_3_](ClO_4_)_2_	2.5	224	210	13	223
	5	235			
	10	243			

**Table 4:** The analysis of XRD data for the Ni-2En-NO_3_, Ni-3En-NO_3_ and Ni-3En-ClO_4_ samples.

Sample	Phase	Content, wt%	CSR Size from Lvol-IB/Lvol-FWHM, nm
Ni-2En-NO_3_	NiO	65	16 ± 1/22 ± 1
	Ni	35	43 ± 1/60 ± 1
Ni-3En-NO_3_	NiO	50	16 ± 1/22 ± 1
	Ni	50	41 ± 1/53 ± 1
Ni-3En-ClO_4_	NiO	-	25 ± 1/36 ± 2

**Table 5:** Elemental composition of the catalyst surface for the Ni-2En-NO_3_, Ni-3En-NO_3_ and Ni-3En-ClO_4_ samples (XPS data).

Sample	Content, at%				
	Cl	C	O	Ni	N
Ni-2En-NO_3_	0	27.2	38.0	34.3	0.5
Ni-3En-NO_3_	0	23.4	39.2	37.1	0.3
Ni-3En-ClO_4_	20.6	44.5	13.8	12.5	8.6

## Data Availability

Not applicable.

## Supplementary Materials

The following supporting information can be downloaded at https://www.mdpi.com/article/10.3390/ma16072616/s1: Table S1: The solid-state reaction models [1,2]; Figure S1: Models of thermal decomposition of (a) [Ni(C_2_H_8_N_2_)_2_](NO_3_)_2_, (b) [Ni(C_2_H_8_N_2_)_3_](NO_3_)_2_ and (c) [Ni(C_2_H_8_N_2_)_3_](ClO_4_)_2_ complexes in helium with a heating rate of 5 °C·min^−1^ (5 mg, helium, 20 mL·min^−1^, 5 °C·min^−1^); Figure S2: DSC data for (a) [Ni(C_2_H_8_N_2_)_2_](NO_3_)_2_, (b) [Ni(C_2_H_8_N_2_)_3_](NO_3_)_2_ and (c) [Ni(C_2_H_8_N_2_)_3_](ClO_4_)_2_ complexes in helium at different heating rates (5 mg, helium, 20 mL·min^−1^); Figure S3: FTIR spectra of Ni-2En-NO_3_, Ni-3En-NO_3_, Ni-3En-ClO_4_ and g-C_3_N_4_; Figure S4: Mass spectrometry (DMSTA) of gas released during the thermal decomposition of (a) [Ni(C_2_H_8_N_2_)_2_](NO_3_)_2_, (b) [Ni(C_2_H_8_N_2_)_3_](NO_3_)_2_ and (c) [Ni(C_2_H_8_N_2_)_3_](ClO_4_)_2_ complexes (5 mg, argon, 100 mL·min^−1^); Figure S5: XPS survey spectra for Ni-2En-NO_3_, Ni-3En-NO_3_ and Ni-3En-ClO_4_ samples; Figure S6: FTIR spectra of the gas mixture at the reactor outlet during the activation of the industrial NIAP-07-01 catalyst at different temperatures; Figure S7: FTIR spectra of the gas mixture at the reactor outlet at 450 °C over the samples obtained from complexes: Ni-3En-ClO_4_—tris(ethylenediamine)nickel(II) perchlorate; Ni-6Im-ClO_4_—hexa(imidazole)nickel(II) perchlorate; Ni-6Im-NO_3_—hexa(imidazole)nickel(II) nitrate; Ni-3En-NO_3_—tris(ethylenediamine)nickel(II) nitrate; Ni-2En-NO_3_—bis(ethylenediamine)nickel(II) nitrate. References [63,64] are cited in Supplementary Materials.
