# Supplementary material for: CO2 Methanation: Solvent-Free Synthesis of Nickel-Containing Catalysts from Complexes with Ethylenediamine
Source: Materials (Basel). 2023 Mar 25;16(7):2616. doi: 10.3390/ma16072616 (PMC10095988; doi:10.3390/ma16072616)
Supplement: Supplementary file 1 [file materials-16-02616-s001.zip › materials-2278193-supplementary.pdf]

SUPPLEMENTARY MATERIAL

CO_2 methanation: Solvent-Free Synthesis of Nickel -Containing Catalysts from Complexes with Ethylenediamine

Olga V. Netskina [1,*], Kirill A. Dmitruk [1,2], Olga I. Mazina [1], Alexander A. Paletsky [1,3], Svetlana A. Mukha [1], Igor P. Prosvirin [1], Alena A. Pochtar [1], Olga A. Bulavchenko [1], Andrey G. Shmakov [3], Janna V. Veselovskaya [1,2], Oxana V. Komova [1]

[1] Boreskov Institute of Catalysis SB RAS, Pr. Akademika Lavrentieva 5, 630090 Novosibirsk, Russia
[2] Novosibirsk State University, 1 Pirogova Str., Novosibirsk, 630090, Russia
[3] Voevodsky Institute of Chemical Kinetics and Combustion SB RAS, 3 Institutskaya Str., 630090 Novosibirsk, Russia
* Correspondence: netskina@catalysis.ru (O.V.N.); Tel.: +7-383-330-74-58

Table S1. The solid-state reaction models [1,2].

	Model	Differential form f(α)	Integral form g(α)
Reaction order model			
1	Mampel	$1 - \alpha$	$-\ln(1-\alpha)$
Diffusion models			
2	1D Diffusion	$(1/2) \cdot \alpha$	α^2
3	Jander (3D Diffusion)	$(3/2) \cdot (1-\alpha)^{2/3}/[1-(1-\alpha)^{1/3}]$	$[1-(1-\alpha)^{1/3}]^2$
4	Ginstling-Brounshtein (3D Diffusion)	$(3/2)/ [(1-\alpha)^{-1/3}-1]$	$1-(2\alpha/3)-(1-\alpha)^{2/3}$
Geometrical contraction models			
5	Contracting cylinder	$2(1-\alpha)^{1/2}$	$1-(1-\alpha)^{1/2}$
6	Contracting sphere	$3(1-\alpha)^{2/3}$	$1-(1-\alpha)^{1/3}$
Sigmoidal models			
7		$2\alpha^{1/2}$	$\alpha^{1/2}$
8	Power law	$3\alpha^{2/3}$	$\alpha^{1/3}$
9		$4\alpha^{3/4}$	$\alpha^{1/4}$
10		$2(1-\alpha) \cdot [-\ln(1-\alpha)]^{1/2}$	$[-\ln(1-\alpha)]^{1/2}$
11	Avrami-Erofeev	$3(1-\alpha) \cdot [-\ln(1-\alpha)]^{2/3}$	$[-\ln(1-\alpha)]^{1/3}$
12		$4(1-\alpha) \cdot [-\ln(1-\alpha)]^{3/4}$	$[-\ln(1-\alpha)]^{1/4}$

1. Nyazika, T.; Jimenez, M.; Samyn, F.; Bourbigot, S. Pyrolysis Modeling, Sensitivity Analysis, and Optimization Techniques for Combustible Materials: A Review. *J. Fire Sci.* 2019, *37*, 377–433, doi:10.1177/0734904119852740.
2. Khawam, A.; Flanagan, D.R. Solid-State Kinetic Models: Basics and Mathematical Fundamentals. *J. Phys. Chem. B* 2006, *110*, 17315–17328, doi:10.1021/jp062746a

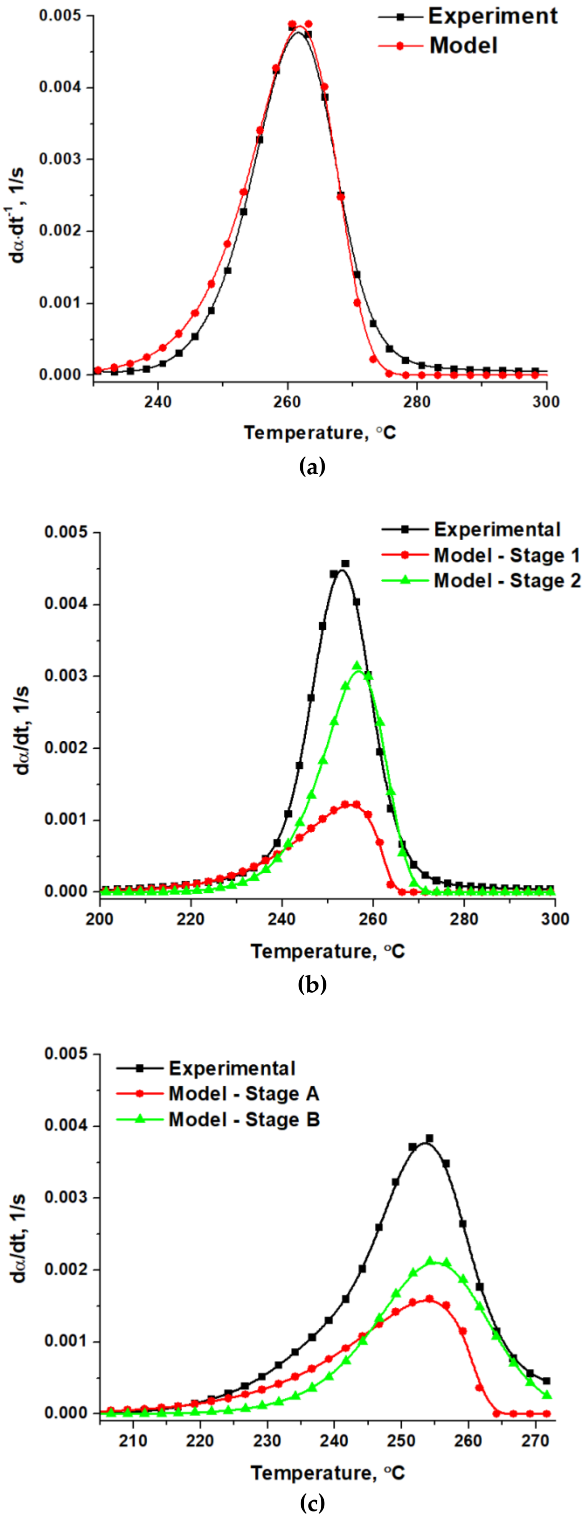

Figure S1. Models of thermal decomposition of **(a)** $[Ni(C_2H_8N_2)_2](NO_3)_2$, **(b)** $[Ni(C_2H_8N_2)_3](NO_3)_2$ and **(c)** $[Ni(C_2H_8N_2)_3](ClO_4)_2$ complexes in helium with a heating rate of 5 °C·min^{-1} (5 mg, helium, 20 mL·min^{-1}, 5 °C·min^{-1}).

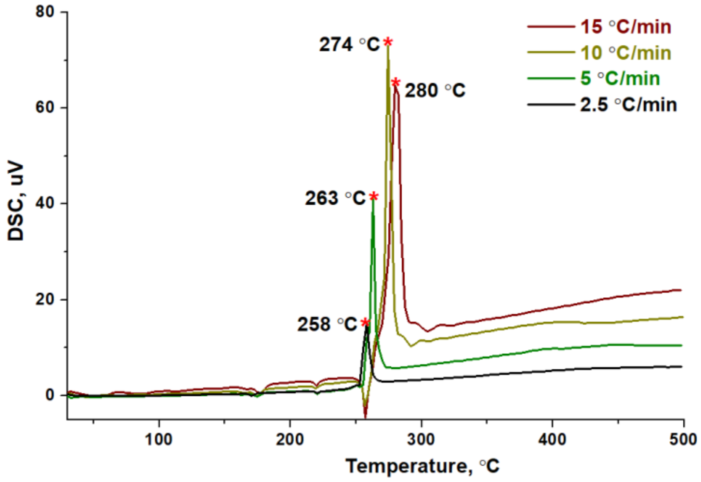

(a)

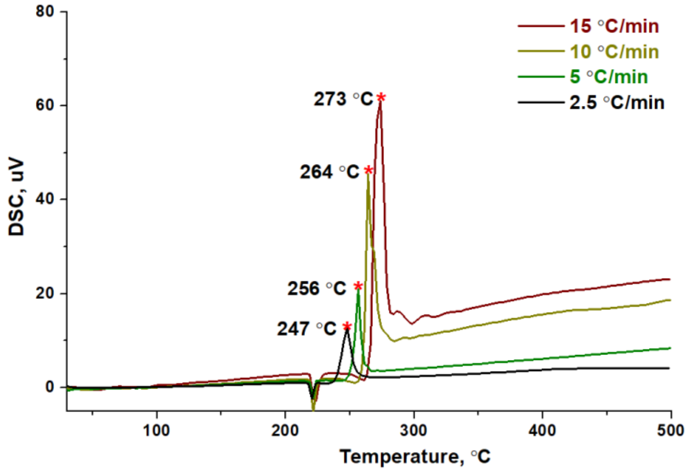

(b)

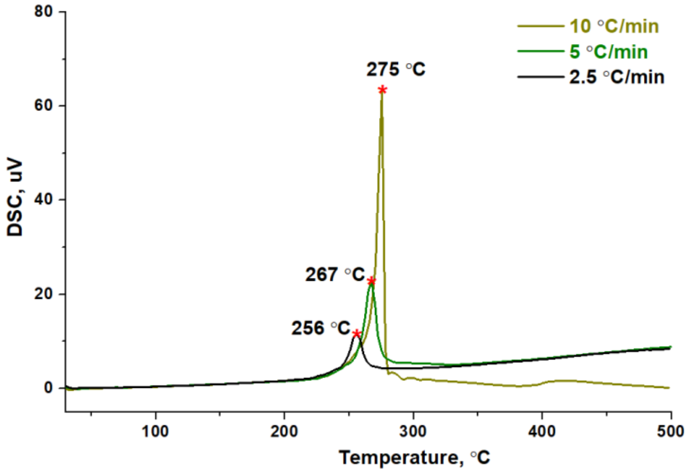

(c)

Figure S2. DSC data for **(a)** $[Ni(C_2H_8N_2)_2](NO_3)_2$, **(b)** $[Ni(C_2H_8N_2)_3](NO_3)_2$ and **(c)** $[Ni(C_2H_8N_2)_3](ClO_4)_2$ complexes in helium at different heating rates (5 mg, helium, 20 mL·min^{-1}).

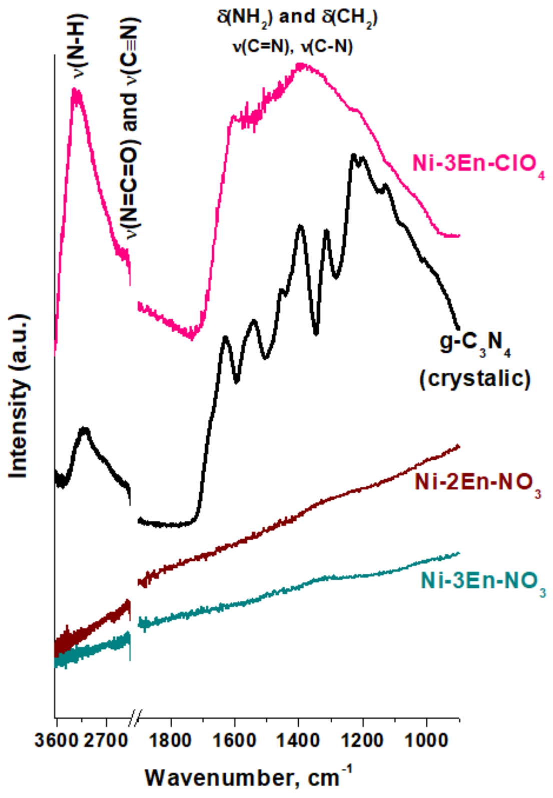

Figure S3. FTIR spectra of Ni-2En-NO_3, Ni-3En-NO_3, Ni-3En-ClO_4 and g-C_3N_4.

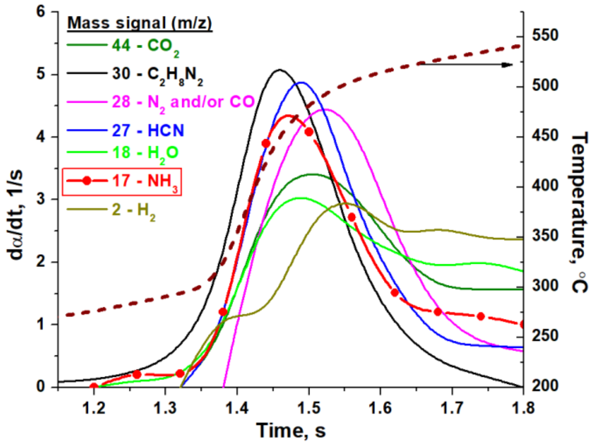

(a)

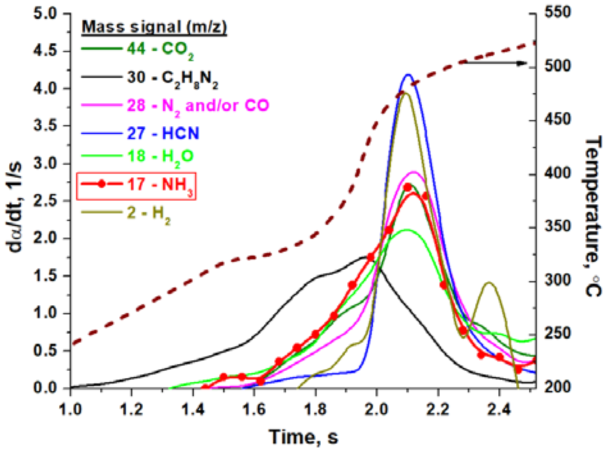

(b)

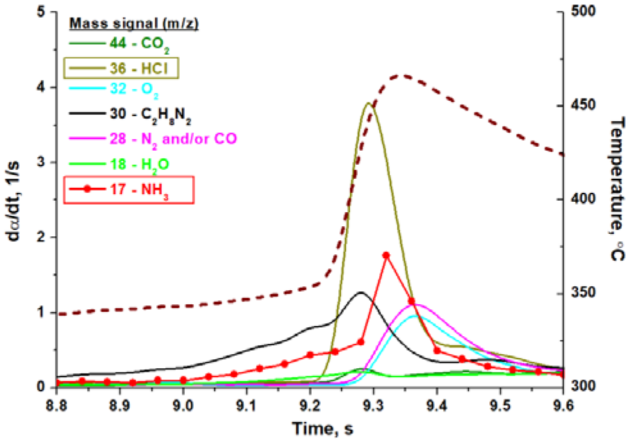

(c)

Figure S4. Mass spectrometry (DMSTA) of gas released during the thermal decomposition of **(a)** [Ni($C_2H_8N_2$)$_2$](NO_3)$_2$, **(b)** [Ni($C_2H_8N_2$)$_3$](NO_3)$_2$ and **(c)** [Ni($C_2H_8N_2$)$_3$](ClO_4)$_2$ complexes (5 mg, argon, 100 mL·min^{-1}).

Method description

The composition of gaseous products of combustion was analyzed by the dynamic mass-spectral thermal analysis (DMSTA) method, using a time-of-flight mass spectrometer with a molecular beam sampling system MSCh-4 (Plant Of Scientific Instrumentation, Sumy, USSR) under a flow of Ar (5 mL·min^{-1}). Average heating rate was ~300 °C·s^{-1}. The sample weight was 1-5 mg. The delay between measurements was 0.04 s. The identification of mass spectral signals was carried out using the mass spectra of individual substances from the NIST database.

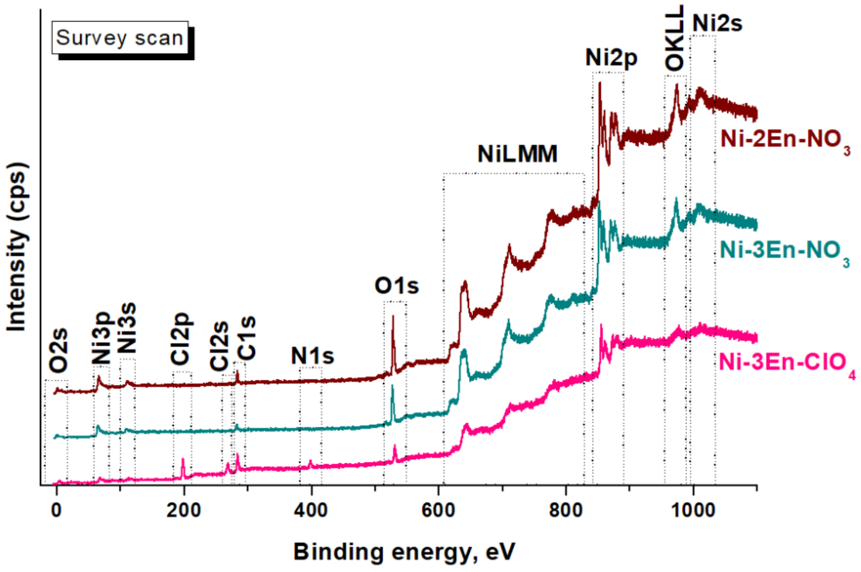

Figure S5. XPS survey spectra for Ni-2En-NO_3, Ni-3En-NO_3, Ni-3En-ClO_4 samples.

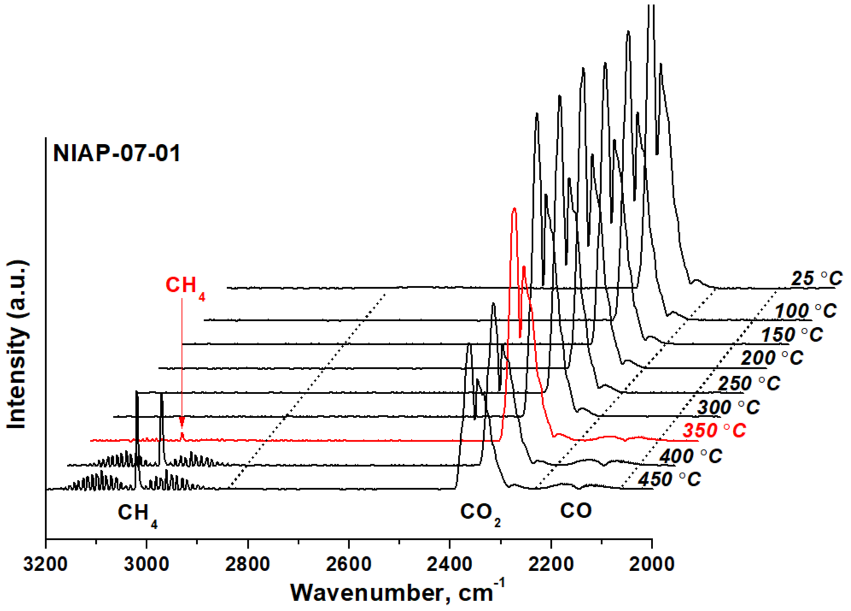

Figure S6. FTIR spectra of the gas mixture at the reactor outlet during activation of the industrial NIAP-07-01 catalyst at different temperatures.

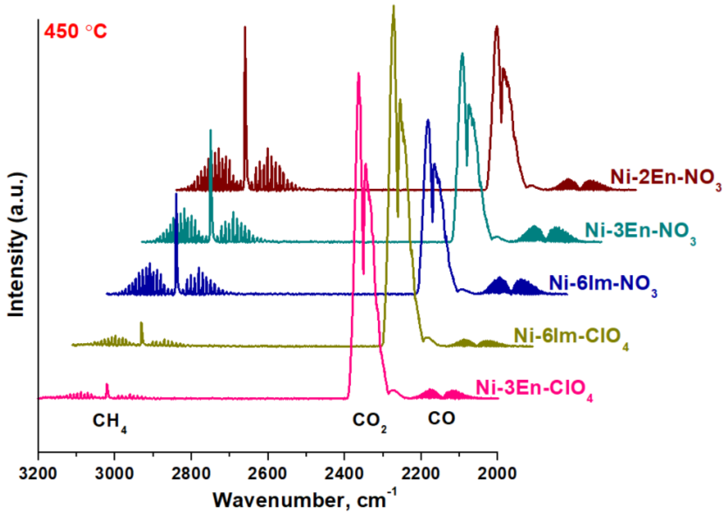

Figure S7. FTIR spectra of the gas mixture at the reactor outlet at 450 °C over the samples obtained from complexes: Ni-3En-ClO_4 – tris(ethylenediamine)nickel(II) perchlorate; Ni-6Im-ClO_4 – hexa(imidazole)nickel(II) perchlorate; Ni-6Im-NO_3 – hexa(imidazole)nickel(II) nitrate; Ni-3En-NO_3 – tris(ethylenediamine)nickel(II) nitrate; Ni-2En-NO_3 – bis(ethylenediamine)nickel(II) nitrate.
